# Using chronobiology-based second-generation artificial intelligence digital system for overcoming antimicrobial drug resistance in chronic infections

**DOI:** 10.1080/07853890.2022.2163053

**Published:** 2023-01-03

**Authors:** Yotam Kolben, Henny Azmanov, Ram Gelman, Danna Dror, Yaron Ilan

**Affiliations:** a Department of Medicine, Faculty of Medicine, Hadassah Medical Center, Hebrew University, Jerusalem, Israel; b Department of Clinical Microbiology and Infectious Diseases, Faculty of Medicine, Hadassah Medical Center, Hebrew University, Jerusalem, Israel

**Keywords:** Digital health, drug resistance, chronic infections, chronobiology

## Abstract

Antimicrobial resistance results from the widespread use of antimicrobial agents and is a significant obstacle to the effectiveness of these agents. Numerous methods are used to overcome this problem with moderate success. Besides efforts of antimicrobial stewards, several artificial intelligence (AI)-based technologies are being explored for preventing resistance development. These first-generation systems mainly focus on improving patients’ adherence. Chronobiology is inherent in all biological systems. Host response to infections and pathogens activity are assumed to be affected by the circadian clock. This paper describes the problem of antimicrobial resistance and reviews some of the current AI technologies. We present the establishment of a second-generation AI chronobiology-based approach to help in preventing further resistance and possibly overcome existing resistance. An algorithm-controlled regimen that improves the long-term effectiveness of antimicrobial agents is being developed based on the implementation of variability in dosing and drug administration times. The method provides a means for ensuring a sustainable response and improved outcomes. Ongoing clinical trials determine the effectiveness of this second-generation system in chronic infections. Data from these studies are expected to shed light on a new aspect of resistance mechanisms and suggest methods for overcoming them.

IMPORTANCE SECTION

The paper presents the establishment of a second-generation AI chronobiology-based approach to help in preventing further resistance and possibly overcome existing resistance.

**Key messages**
- Antimicrobial resistance results from the widespread use of antimicrobial agents and is a significant obstacle to the effectiveness of these agents.
- We present the establishment of a second-generation AI chronobiology-based approach to help in preventing further resistance and possibly overcome existing resistance.

## Introduction

In 1928, penicillin was discovered, and it took more than a decade for the drug to be widely used [1]. Penicillin is still a cornerstone in the antimicrobial field almost a century later, although significant problems have emerged. Penicillinase, which inactivates penicillin, was identified before the commercial use of penicillin, suggesting an inherent property of some bacteria which have evolved over millennia preceding its discovery by humankind [2]. Numerous antibiotic agents were developed, and accordingly, mechanisms of resistance evolved [3]. More than 20,000 potential resistance genes (r genes) were identified [4].

Nevertheless, although new antibiotics are being studied, the last antibiotics class was discovered in 1987, with Lipopeptides, specifically daptomycin [5,6]. With the rising prevalence of antibiotic resistance and the development of multidrug-resistant (MDR) strains, better strategies to overcome drug resistance are needed. Targeting the bacteria’s antimicrobial resistance mechanism is widely used, such as beta-lactamase inhibitors [7]. Recent advances focus on targeting biofilms, quorum sensing, and other virulence factors [8] and education for more judicious use of antibiotics [9].

The development of resistance is a significant obstacle to antiviral therapy. Most active antiviral agents have been shown to select resistance mutations [10]. Virus replication generates a large number of quasispecies. Viral resistance has implications for treatment regimens and the design of new therapeutics [11].

The cyclical nature over a 24-h period is a fundamental feature conserved across most life on earth. Processes are compartmentalized concerning time in a manner that mirrors the rotation of the planet and accompanying diurnal cycles of light and darkness [12]. Chronobiology is the study of biological rhythms, which examines the effects of time on biological events and internal biological clocks [13]. Chronobiology is inherent in all biological systems. Host response to infections and pathogens activity are affected by the circadian clock [14].

The paper reviews the relevant papers describing the mechanisms associated with drug resistance in chronic infections. We reviewed the relevant studies using digital platforms to deal with this problem, mainly by reminding patients to adhere to their treatments. It presents the establishment of a second-generation artificial intelligence (AI) chronobiology-based approach which implements personalized variability signatures to help prevent and possibly overcome existing resistance.

## The problem of drug resistance in chronic infection and the role of chronobiology in the pathogenesis of infections

Several infections are prone to become chronic. The biological processes leading to cure failure at the acute stage, progression to chronicity, and lack of response to drugs are multifactorial, poorly understood, and associated with worse prognoses [15–18].

Numerous antimicrobial resistance mechanisms affect a wide variety of antimicrobial agents [19]. Following are prime examples of resistance-limited effective therapies for infectious diseases that often persist despite initial antimicrobial-active therapy:

Tuberculosis (TB) is the leading cause of death from an infectious disease. It is estimated that 1.7 billion people around the globe are infected with *Mycobacterium tuberculosis*, and reactivated disease is the most common manifestation in the elderly and immunocompromised patients [20]. Drug-resistant TB accounts for 4–6% of TB cases and exceeds 25% in some areas, especially former soviet-union countries and India [21]. The proposed solutions for MDR TB include prolonged courses of up to seven different agents associated with a high incidence of side effects, 45% of which are moderate to severe [22].

*Staphylococcus aureus* (*S. aureus*) is the leading pathogen causing acute and chronic osteomyelitis. It produces biofilm, which increases bacterial antibiotic tolerability by up to 1000-fold [23]. The mechanisms by which biofilm affects antibiotic resistance are not well understood, as some antibiotics can diffuse well through the extracellular matrix of the biofilm [24]. Additional factors leading to treatment failure exist, including altered metabolic activity, slow bacterial growth, and intracellular survival of *S. aureus* [25].

Chronic rhinosinusitis is one of the more prevalent chronic illnesses that evolves following the acute phase and is challenging to treat. Few of the commonly involved bacteria can appear in their drug-resistant form. For example, 20% of *S. aureus* species are methicillin-resistant, and *Pseudomonas aeruginosa* isolates showed a 27% resistance rate to quinolones and a 36% resistance rate to aminoglycosides [26].

The World Health Organization has recognized Clarithromycin-resistant *Helicobacter pylori* as a ‘high priority’ issue, requiring new antibiotics. Resistance to metronidazole is also emerging. Tetracycline and amoxicillin resistance rates are still low but rising [27]. These reported resistances led to profound treatment failures. When strains are resistant to clarithromycin, the eradication failure rate is as high as 88%, even if the bacteria are susceptible to other antibiotics in the regimen, while when strains are susceptible, the failure rate is 18% [28]. In this setting, eradication failure may lead to severe complications, such as gastric cancer and peptic ulcer [29].

These chronic infections represent some clinical challenges where initial failed therapy and rising antibiotic resistance rates complicate effective therapy. It is plausible that other host- and pathogen-related factors could enhance the effectiveness of antimicrobial treatment, thus limiting progression to chronicity.

Chronobiology examines timing processes, including periodic phenomena in biological systems and their adaptation to solar and lunar rhythms. These cycles are known as biological rhythms [14,30,31]. Chronobiology plays a role in the function of multiple organs, including the immune system. Several inflammatory diseases, such as rheumatoid arthritis, myocardial infarctions, and ischemic stroke, have shown diurnal variation [32].

The molecular clock’s critical aspects also control the immune system’s properties. The suprachiasmatic nucleus (SCN) is responsible for maintaining circadian rhythm. Its function is based on several transcription-translation feedback loops. The SCN also synchronizes the peripheral clocks found in most systems *via* the hypothalamic-pituitary-adrenal axis and the autonomic nervous system (ANS) [33].

Several vital proteins, including CLOCK (Circadian locomotor output cycles kaput), BMAL1 [Brain and muscle aryl hydrocarbon receptor nuclear translocator (ARNT)-like 1], and REV-ERB α, have been implicated in immune function [33,34]. BMAL1 affects chemokines and can suppress them, thereby exerting an anti-inflammatory effect. REV-ERB α affects interleukin-6 and macrophage activity [32]. The role of CLOCK is not fully understood, but its overexpression has been shown to increase the activity of the NF-k B complex [33].

The number of mature circulating leukocytes is higher during the resting phase, and mature cells are released from the bone marrow at the commencement of this phase [34]. Cytokine release is affected by the circadian rhythm, increasing proinflammatory cytokines in the resting phase. The circadian nature of the immune system impacts the response to vaccines. Studies on hepatitis A and influenza immunization showed higher antibody titers for those vaccinated in the morning [35].

Numerous parasites, viruses, bacteria, and gut microbiome species manifest circadian rhythms [14]. Studies in mice infected with *Listeria monocytogenes* showed that mice infected at the beginning of the rest phase have a higher colonization burden than at the end of the rest phase [36]. Alternatively, mice infected with subspecies of *Salmonella enterica* have a higher bacterial load when infected in the rest phase than in the active phase [37]. Studies done with *Streptococcus pneumoniae*, and *Chlamydia muridarum* also show circadian variation [38]. The host’s circadian rhythm affects the gut microbiome, while some species, such as *Klebsiella aerogenes*, have an intrinsic rhythm [38]. Last, for parasites, replication, and transmission of malaria, a pathogen with a high death toll, is negatively affected when not in sync with the host’s circadian rhythm [39].

Increasing evidence suggests that bacteria have a circadian rhythm that may affect antibiotic resistance [14]. Studies on *S. aureus* and clinically significant Enterobacteriaceae showed minimal inhibitory concentration (MIC) changes at certain times [40,41]. A study on cystic fibrosis patients showed that when Ceftazidime was administered in the morning, the blood’s drug concentration was higher compared to the afternoon [42]. The administration of antibiotics at certain times of day can also be relevant to reducing adverse effects. For example, when aminoglycosides are administered during the resting phase, their nephrotoxicity is greatest [43].

The circadian clock regulates various aspects of viral replication, host responses, and associated pathogenesis. A disrupted circadian clock has been linked to increased susceptibility to several viral-associated diseases. Viruses are obligate parasites reliant on their hosts for replication and dissemination. As more than 80% of protein-coding genes in various tissues show daily rhythmic expression, and given the dependency of virus replication on cellular pathways, host clock components directly or indirectly influence virus replication [44]. Numerous factors that synchronize peripheral clocks, including the autonomic nervous system, body temperature, fasting/feeding cycles, and cytokines and hormones, may impact viral-mediated pathologies [44]. Viruses can reprogramme cellular metabolism, affecting the mechanisms involved in circadian rhythm [45]. Chronobiology is important for designing therapeutic strategies against SARS-CoV-2 and COVID-19, and other viruses [46].

## Using first-generation digital platforms for improving the effectiveness of anti-infectious agents

The use of digital platforms has steadily increased over the last decade [47]. These include systems that analyze data for assisting in diagnosis and treatment and apps provided to patients to remind them to take their medications [48,49]. With the growing use of cellular phones and smartphones and the expanding coverage of mobile networks in low-resource and high-resource countries, an advancement was made in adapting digital platforms to various diseases and medical conditions [50]. A significant adaptation of such is the development of multiple digital adherence technologies (DAT) [51] in patients with human immunodeficiency virus (HIV) [52], hepatitis C virus (HCV) [53], and TB [51].

Numerous studies in Africa and Asia have examined short messaging service (SMS) automated texts sent to patients’ family members’ phones, reminding them to take medications. In several intervention studies, patients could respond by returning an SMS or placing a telephone call when the task was performed. Such an intervention has a well-established adherence improvement in HIV and TB [52,54–56].

Several studies assessed instant live-streaming video conference calls allowing health care providers to perform a directly observed therapy (DOT) from a distance watching the patient take his/her medications at home. It was primarily used in high-income countries, such as Singapore [57], United States [58], and Mexico [59].

Medication event and reminder monitoring consistent with digital pillboxes with a pre-determined reminder to take medications can improve patient adherence. Opening and closing the box is used as a digital indicator for regimen adherence and is stored on internal memory allowing the health care provider to access the dosing history created by the patient [60,61].

A novel platform comprises ingestible sensors implanted in medications for TB, HCV, HIV, and other medical conditions where adherence is crucial to avoid significant morbidity and mortality [62]. Upon ingestion, the sensor interacts with the patient’s gastric fluid sending a signal transmitted to a monitor adhered to the patient’s skin, which sends a signal to the patient’s smartphone allowing sharing of the information with the patient’s physician.

These technologies, while improving adherence, do not assist in overcoming drug resistance.

## Implementing a second-generation AI chronobiology-based regimens for overcoming drug resistance in infections

Implementing variability in administration times and dosing regimens was proposed to overcome resistance to chronic drugs [63–74]. Drug holidays and dosage modifications were shown to improve clinical response to drugs [75–80]. Drug holidays were evaluated while using chronic anti-infection agents [81].

Preventing virological failure following HIV treatment is complicated by the emergence of drug resistance. A mathematical model that predicts HIV virological response toward various compounds was developed. The model is based on drug penetration into lymph nodes, refined adherence, pharmacokinetic and pharmacodynamic variability, drug interaction, and cross-resistance. The model showed that limited lymph node drug penetration accounts for a large proportion of virological failure and drug resistance [82].

A stochastic model was developed that incorporates compartments of latently infected cells and virus genotypes with different drug susceptibilities. Parameters that promoted resistance included a high reproductive number, extended drug holidays, and poor adherence. The model supports the importance of the interactions between imperfect adherence, pharmacodynamics, pharmacokinetics, and latently infected cells as factors in therapy failure in HIV anti-retroviral therapy [83].

Models were developed for using drug holidays to overcome drug resistance to infections. There is a nongenetic variation in phenotypes in bet-hedging that induces drug resistance in both bacterial infections. A model was developed to determine how bet-hedging emerges *via* a molecular switch in genotype-phenotype (GP) mapping. The model used stochastic switches that map gene products to phenotypes. Bet-hedging emerged in this model and is robust to evolutionary loss through mutations to both the expression of individual genes and the network itself. The data support the use of treatment holidays to combat bet-hedging-driven resistant disease. It implies that the structure of the GP mapping can determine the efficacy of treatment breaks [84].

A digital system was developed for implementing variability and chronobiology-based regimen for overcoming drug resistance and improving the effectiveness of chronic medications [30,31,65,68,69,85–100]. Using variability to improve organ function was proposed based on studies showing its fundamental role in complex biological systems [64,70–73,99–105]. This second-generation digital system overcomes some of the major obstacles associated with the lack of penetration of first-generation systems in everyday clinical practice [74,106,107].

The second-generation system targets a clinically meaningful endpoint that ensures adaption by caregivers and adherence by patients. Thus, rather than just reminding patients to take the drug, which is inherent to most first-generating platforms, both patients and physicians view the system as part of the therapy, as they can follow up on the clinical effect. While large datasets are mandatory for most first-generation algorithms, the second-generation system implements the *n* = 1 concept, thus overcoming the hurdles of using unclean datasets that affect the algorithm outputs [74,106,107].

The system is being developed in three steps [107]. In the first step, the algorithm randomizes the administration times within the approved pharmacokinetic range. The algorithm can also randomize the dose administrated within the regulatory-approved range. Introducing variability is expected to prevent the development of compensatory mechanisms associated with drug resistance [74].

In the second step, the algorithm personalizes the regimen using a closed-loop system that uses clinical and laboratory endpoints. At this step, individualized chronotherapy-based responses are being implemented, enabling further drug improvement. Using clinically meaningful endpoints adapts the algorithm to optimize the therapeutic regimen [74,106,107].

In the third step, implementing signatures of variability, which are relevant to the chronobiology of the infections agents, or the host response, are implemented into the algorithm. The learning algorithm develops chronobiology signatures by identifying the ideal timing for administering an anti-infective agent in correlation with host, disease, and virulence factor-related outcomes. Additional signatures comprise ANS variables relevant to chronobiology, such as heart rate variability [20,69]; variability is pro and anti-inflammatory cytokines that determine the host response towards the infectious agent [64,92]; and modifications in the infectious agent epitopes and genes.

In summary, drug resistance is a significant obstacle to the use of most drugs, mainly in patients who require chronic therapeutic regimens. An algorithm-controlled regimen that improves the long-term effectiveness of anti-infectious agents is being developed. It provides a means for ensuring a sustainable response and improved outcomes. Ongoing clinical trials determine the effectiveness of second-generation systems in chronic diseases, including chronic infections. Data from these studies are expected to shed light on the resistance mechanism to anti-infectious agents and methods for overcoming them.

## Data Availability

All data is on public domains.
